# Tau in Oligodendrocytes Takes Neurons in Sickness and in Health

**DOI:** 10.3390/ijms19082408

**Published:** 2018-08-15

**Authors:** Patrizia LoPresti

**Affiliations:** Department of Psychology, University of Illinois at Chicago, 1007 West Harrison Street, Chicago, IL 60607, USA; lopresti@uic.edu

**Keywords:** microtubules, multiple sclerosis, oligodendrocytes, tau, myelin

## Abstract

Oligodendrocytes (OLGs), the myelin-forming cells of the central nervous system (CNS), are lifelong partners of neurons. They adjust to the functional demands of neurons over the course of a lifetime to meet the functional needs of a healthy CNS. When this functional interplay breaks down, CNS degeneration follows. OLG processes are essential features for OLGs being able to connect with the neurons. As many as fifty cellular processes from a single OLG reach and wrap an equal number of axonal segments. The cellular processes extend to meet and wrap axonal segments with myelin. Further, transport regulation, which is critical for myelination, takes place within the cellular processes. Because the microtubule-associated protein tau plays a crucial role in cellular process extension and myelination, alterations of tau in OLGs have deleterious effects, resulting in neuronal malfunction and CNS degeneration. Here, we review current concepts on the lifelong role of OLGs and myelin for brain health and plasticity. We present key studies of tau in OLGs and select important studies of tau in neurons. The extensive work on tau in neurons has considerably advanced our understanding of how tau promotes either health or disease. Because OLGs are crucial to neuronal health at any age, an understanding of the functions and regulation of tau in OLGs could uncover new therapeutics for selective CNS neurodegenerative diseases.

## 1. Introduction

Oligodendrocytes (OLGs) play a critical role in central nervous system (CNS) health or disease due to their closely linked partnership with neurons [1,2,3]. OLGs, classically connected to myelination, mostly during the postnatal period when many neuronal functions (e.g., walking and cognition) become possible, are now considered essential for adjusting to neuronal functions. Indeed, OLGs become tuned in to the visuomotor skill learning and social behaviors occurring in the young, adult, and old [2,4,5,6], resulting in a myelin that can adapt; new OLGs and internodes are generated to adjust to the lifelong needs of neurons [7,8]. Such ability to adapt to and alter brain functions during the ever-changing lifelong demands is the hallmark of a healthy CNS. By contrast, when such flexibility falls short, the OLG-neuron unit breaks down, and CNS degeneration follows. How OLGs and myelin bring about normal versus pathological neurons must be fully elucidated because it might reveal key events that could be potential pharmacological targets to treat neurodegeneration and cognitive decline.

**Figure 1:**
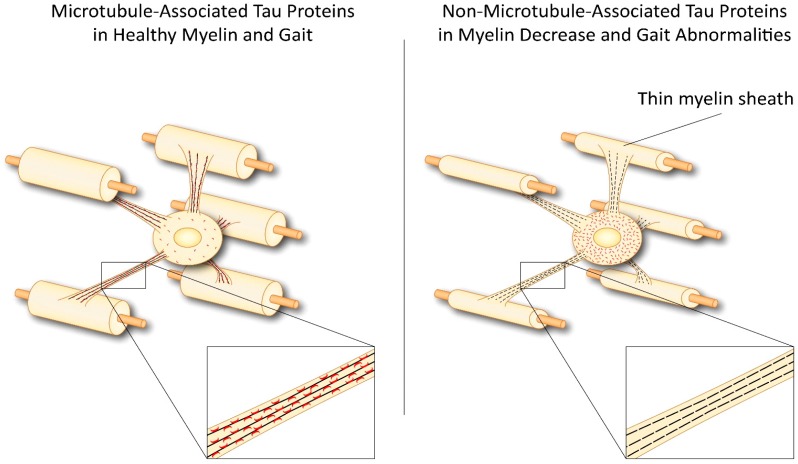
The Sorting of Tau into Oligodendrocyte Processes for Neuronal Health. The key functional property of tau is its association with microtubules. The sorting of tau into OLG processes and process tips fails as a consequence of alterations in splicing mechanisms and posttranslational modifications, or when tau is fragmented. The sorting of tau into OLG processes and process tips enables correct thickness of myelin around the axon, whereas missorting of tau causes a reduction in the thickness of myelin, with deleterious effects on neuronal functions [16]. Red dots represent tau.

The microtubule-associated protein tau is involved in both the health and disease of neurons [9,10]. In OLGs, tau plays a key role in myelination [11,12,13,14], and its malfunction causes myelin and movement disorders [15,16] (Figure 1). It is not surprising that OLG tau can be either beneficial or detrimental, as it regulates events within cellular processes that are essential for cellular process extension and myelin formation [12,13,14,17,18]. Areas of future research must seek to understand the molecular mechanisms by which OLG tau impacts the neurons, and how a deregulation of OLG tau might negatively impact the OLG-neuron unit.

The OLG processes are very long, with the myelin at their tips at a considerable distance from the OLG body. Because a single OLG wraps axonal segments of distinct neurons, many processes from a single OLG can be locally regulated at their tips by mechanisms governed by the neuronal subtype and function [2]. The regulation of tau in OLGs can indeed be tailored to neuronal subtypes, axonal identity, and function. Several mechanisms may accomplish this task. Growth factors, cytokines, and neurotransmitters are all important for tau regulation in OLGs; however, more work is required to understand their specific effects on tau in OLGs.

Recent studies have shown that *N*-Methyl-d-aspartate (NMDA) receptors sustain functional OLG-neuron units [2]. In particular, activation of oligodendroglia NMDA receptors triggers glucose transporter membrane insertion, which increases OLG glucose uptake capacity and consequent lactate release into the axonal compartment to accommodate the high energy demands of healthy axons [2]. One question for future research is whether the tau–non-receptor-associated tyrosine kinase Fyn interaction in OLGs might regulate NMDA receptors in these cells. The interaction of tau with Fyn in OLGs is pivotal for myelination and healthy neurons [15,19]. The NMDA receptor consists of three different subunits: NR1, NR2, and NR3. Previous work showed that NMDA receptors are present in the myelinating processes of oligodendrocytes [20]; among the various subunit isoforms, NR1, NR2C, and NR3A mRNA are abundant in whole optic nerve. NR2B mRNA is present at low abundance, whereas NR3B is absent [21,22]. It is known that the postsynaptic NR subunit NR2b is a substrate of Fyn [23,24], and Ittner et al. [25] showed that Y1472 phosphorylation is significantly reduced when Fyn is not targeted to the process tips, due to the expression (in neurons) of a truncated form of tau with no microtubule-binding domain. Future studies will determine whether an alteration of the tau–Fyn interaction, or more generally of tau in OLGs, causes CNS degeneration due to an alteration of OLG NMDA activation and diminished lactate entering the neurons. Such a decrease would indeed be quite deleterious for the axons and potentially accounts for the CNS degeneration driven by a malfunction of tau in OLGs.

Previous studies showed that excitotoxicity lesions decrease myelin basic protein levels [26], and that the intracervical perfusion of glutamate increases tau immunoreactivity specifically in OLGs [27]. Although it is well established that modulation of NMDA receptors regulates tau phosphorylation, additional studies must be conducted to gain insight into the effects on tau, specifically in OLGs. Within the context of the interplay between OLGs and neurons, OLG tau must be viewed as the gatekeeper of a healthy OLG-neuron unit.

Growth factors are known to offer neuroprotection, and Neu differentiation factor (NDF), an axon-derived factor, is important for myelin regulation. LoPresti et al. [17] showed that NDF added to cultured OLGs increases cellular process extension, and tau protein and mRNA levels. Additional studies showed the importance of other axon-derived signals in myelination [28]. Furthermore, adenosine derived from ATP released from electrically active axons stimulates the morphological differentiation of OLG precursor cells [29].

Myelin biogenesis requires highly regulated sorting mechanisms [30]. The sorting of tau is particularly important in neurons. Separate tau isoforms are known to reach distinct intracellular sites in neurons and tau distribution is regulated during development [9,31]. As axons are established during neuronal morphological maturation, large quantities of tau localize in the axons, whereas tau remains at very low quantities in dendrites and nuclei. The polarized distribution of tau in neurons is believed to be the result of specialized mechanisms that take place at the level of mRNA and/or tau protein, although the precise mechanism of this selective sorting is unknown [9]. The sorting of tau is altered early in the cascade of events that lead to neurodegeneration; indeed, it is believed that tau sorting anomalies are one of the first defects during Alzheimer’s disease (AD) [9].

In contrast to neurons with functionally distinct processes like axons and dendrites, OLG processes are all myelin-forming processes, so understanding tau sorting in the processes of these cells requires the answering of separate questions about how distinct tau isoforms might influence the sorting mechanisms that regulate protein and mRNA critical for myelin biogenesis. Such regulation of tau isoforms in OLGs is likely to be regulated by neurons by a variety of mechanisms. Such regulation would tailor the myelin to neuronal function [2].

The absence of tau sorting into OLG processes, as occurs in the presence of phosphotau, truncated tau, or a diminished tau, alters the sorting mechanism that underlies myelin formation, with deleterious events that promote neurodegeneration (Figure 1).

During myelin biogenesis, membrane expansion, the transport of selected mRNA and proteins, and the cytoskeleton dynamics are all functionally synchronized [30]. These events still occur in adults, yet it remains to be fully elucidated how neurons regulate these intracellular events in OLGs. During myelin formation, selected proteins reach their intracellular targets by the translocation of free polysomal mRNAs to specific loci where translation starts [30]. It was originally proposed that free polysomes were associated with the cytoskeleton [32]. By and large, because tau protein can associate with the cytoskeleton and regulate motor proteins [9,18], tau can be viewed as the protein of choice for attaching to selective mRNA and/or proteins that are critical for the making of myelin, during both developmental and adult myelination. Thus, tau can functionally bridge the cytoskeleton, motor proteins, and myelin formation.

In summary, myelin formation requires tau in OLG processes and process tips during the lifetime of neurons. A functional dissection of the critical determinants of a healthy OLG-neuron unit and of how tau in OLGs works to promote either healthy or sick neurons is critically important. Studies should consider how tau in OLGs protects or undermines neuronal health. The results of these investigations would apply to diseases of myelin, known in developmental and demyelinating disorders, and apply also to dementia and cognitive behavior disorders.

## 2. Mechanisms of Tau Regulation

The sorting of tau into OLG processes and process tips is essential for a healthy myelin. In contrast, missorting of tau causes reduced myelin and axonal degeneration (Figure 1). To understand which levels of tau regulation in OLGs can be targeted, several studies have established the importance of tau mRNA splicing and of tau posttranslational modifications [9,13,33,34,35]. These events are key to understand how tau can promote either CNS health or disease. Tau mRNA splicing is highly regulated and, in a cell-specific pattern [13]. Contrary to the pattern of neuron regulation, which has a developmentally regulated shift in the tau mRNA form expressed in the young versus adult rodent CNS, tau regulation in OLGs is specific to these cells, with the persistent presence in the adult CNS of forms of tau mRNA splicing that are abundant in neonatal neurons. Although the precise function for each splicing form of tau is unknown [9], the persistent expression in the adult CNS of the neonatal form of tau suggests that tau in OLGs (of the adult CNS) keeps cellular processes functionally *young*, which is perhaps an essential feature of the myelin’s adaptability to neuron function and brain plasticity. The balance between various tau isoforms can indeed enable the myelin to respond to changes of neuronal functions during a lifetime. By contrast, an imbalance of the tau isoforms would make the myelin functionally *rigid*, (i.e., not able to adapt to neuronal functions), with a consequent demise of the OLG-neuron unit. The neonatal versus adult forms of tau differ in the number of microtubule (MT)-binding sites (i.e., three versus four sites, respectively), with three sites resulting in less stable binding to the MTs [9]. Previous work showed that OLGs had a lower expression of four-repeat (4R) tau than that in gray matter, and this unique pattern of expression persists in long-term OLG cultures [13,33,35,36]. During OLG differentiation tau protein levels increase, together with a decrease of tau phosphorylation [33,36]. In addition to the functional significance and regulation of the MT-binding region, several studies have also investigated the amino domain of tau protein, whose function has eluded our full understanding.

Regulation at amino domains also presents a pattern of expression regulation that is specific to OLGs [13]. The tau amino domain contains two inserts of 29 amino acids each. These inserts become expressed in adult neurons; OLGs express low amounts of the amino domain with two inserts, which again shows that OLGs have unique tau function requirements [13,33]. Thus, in the OLG-neuron unit, tau is expressed in both cells, but it has a cell-specific pattern of expression, which could provide distinct functions in the cellular processes of the two cell types [13,33]. Because most of this insight has been obtained in rodent models, studies in human OLGs are critical to fully appreciate the role of tau regulation in both health and disease.

The importance of tau in human OLGs has been largely obtained from studying CNS degenerative diseases [37,38,39,40,41,42,43]. Immunohistochemical staining of OLGs in selected tauopathies reveals (in OLGs) ~14 nm diameter tubules in perikarya (coiled bodies) and outer loop processes of myelin sheath (argyrophilic threads) [37,40]. Such structures contain heavily phosphorylated tau at both the proline-rich and MT-binding site, and elevated levels of 4R tau [37,40]. How the increase of 4R tau undermines the essential features of a healthy CNS (i.e., the capacity to adapt to the neuronal function) must be investigated in future studies.

Additional tau regulation occurs via posttranslational modification [44,45,46]. The most studied of which is tau phosphorylation. Indeed, regulation of tau phosphorylation, which is highly controlled during development, negatively modulates the ability of tau to bind the MTs. Increased tau phosphorylation is a hallmark of CNS degeneration. Tau is highly phosphorylated in the young CNS, and its phosphorylation diminishes by approximately one third in the adult CNS [9,47]. In addition, tau phosphorylation levels diminish during in vitro maturation of OLGs, suggesting that healthy OLG processes require a regulated phosphorylation of tau, taking place at specific sites [36].

Upon exposure to a noxious stimulus, such as oxidative stress, the levels of tau phosphorylation in OLGs change rapidly; these cells respond to the deleterious environments present during various CNS degenerative diseases via modification of tau phosphorylation. Oxidative stress is a major mediator of neurodegeneration. Previous studies [48,49] showed that brief exposure to hydrogen peroxide changes the phosphorylation state tau in OLGs. Hydrogen peroxide treatment caused a morphological degeneration of OLGs with loss of cellular processes; this morphological degeneration is preceded by a profound dephosphorylation of tau protein [48]. A similar H_2_O_2_-induced dephosphorylation of tau protein in OLGs was previously observed in neurons during continuous exposure to H_2_O_2_ [50]; the dephosphorylation of tau protein may be an integral part of the cellular response to oxidative stress, and it has been reported following human stroke, head injury, and brain heat shock [51,52,53].

Tau binding to MTs regulates cellular extension and intracellular trafficking [12,14,17,18]; whereas hyperphosphorylation of tau negatively regulates tau binding to MTs and tau functions [9]. In addition to kinases, phosphatases are critical to maintain healthy levels of tau phosphorylation [54]. Although the kinases certainly play a critical role in the abnormal tau phosphorylation observed during CNS degenerative diseases, the activity of protein phosphatase need additional investigation, with emphasis on pharmacological approaches aimed at their regulation. Of interest, in cultured OLGs following oxidative stress, the activation of phosphatases occurs as a very early step [48]. By contrast, such activity is reduced in Alzheimer’s disease, with a stronger reduction in the white matter compared to in the gray matter [9,54]. Thus, the upregulation and downregulation of enzymes that affect tau phosphorylation can result in either CNS protection or damage.

Tau phosphorylation is precisely regulated during development, and its deregulation is believed to be at the center of the pathogenesis of several CNS degenerative diseases [9,55]. Regarding tau phosphorylation, kinases include proline-directed kinases include glycogen synthase kinase 3β (GSK3β), cyclin-dependent kinase 5 (CDK5) and mitogen-activated protein kinase (MAPK) [56,57,58,59]. Of interest, CDK5 positively affects myelination, whereas GSK3β signaling pathways are known to be detrimental [60,61]. Furthermore, inhibitors of GSK3β have been shown to be beneficial in many neuroinflammatory disease models, such as those for Alzheimer’s disease, multiple sclerosis (MS), and AIDS [62].

Kinases involved in CNS degenerative diseases include also the microtubule affinity-regulating kinases (MARKs; known as PAR1 kinases), cyclic AMP-dependent protein kinase (PKA), and Ca^2+^-or calmodulin-dependent protein kinase II (CaMKII) [9,63,64,65]. Dr. Gloria Lee has established the importance of tau phosphorylation at Tyr residues during Alzheimer’s disease [66]. The kinases involved in the process of tau phosphorylation at Tyr residues involve the family of non-receptor tyrosine kinases such as LCK, SYK, and FYN and Abl [67,68,69]. Additional studies are required to understand the functional consequences of tau phosphorylation at Tyr in human OLGs. The deleterious consequences of hyperphosphorylated tau extend to the detachment of tau from MTs and include changes in intracellular transport and tau degradation. As tau detaches from the MTs, it reaches unusual intracellular targets, resulting in detrimental functional outcomes.

Because the CNS can repair itself, the short- versus long-term consequences of acute variations of tau phosphorylation in OLGs are unknown. It is unknown how an acute variation of tau phosphorylation in OLGs might impact CNS health. However, studies of post-traumatic brain injury patients reveal that physical trauma causes long-term consequences, including increased tau phosphorylation as well as myelin and CNS degeneration [70]. Although the consequences of oxidative stress on tau phosphorylation and cellular process outgrowth have been investigated in OLG cultures, future work is required to address short- and long-term consequences of OLG tau post-translation modifications on neurons. Future research could investigate whether pharmacological treatment of tau phosphorylation in OLGs might offer protection from the deleterious outcomes that occur secondary to traumatic or other kinds of brain insults.

In addition to phosphorylation, tau is subject to acetylation and other post-translational modifications, including glycosylation, glycation, deamidation, isomerization, nitration, methylation, ubiquitylation, and sumoylation [9,71,72,73]. The acetylation of tau has been found in the brains of patients with Alzheimer’s disease and other dementias with prominent tau pathology in the OLGs [72]. The acetylation of tau has also been found to support healthy brain functions [9]. Depending on the tau site that is acetylated, thus the consequences of acetylated tau can be either beneficial or deleterious [9].

Tau can also cause a pathology when its proteolytic processing results in tau fragments [74,75]. Such fragments interfere with cellular functions. Tau is a dynamic protein, both structurally and functionally. The ability to fold like a paperclip makes this protein capable of simultaneously binding to distinct cellular partners [9]. The structure of tau has two domains—the amino- and the carboxy-terminal regions. Each domain is separated by a proline-rich domain and has separate intracellular targets and functions. The amino domain binds the membrane, whereas the carboxy domain, called the MT-binding domain, binds the MTs, with the strength MT binding regulated by the number of MT-binding regions. The region of tau between the amino and the carboxy regions is of interest since it binds key signaling pathways critical for tau function in OLGs and myelination [76,77,78].

Tau is known to act as a scaffolding protein for selective signaling, and so altering tau in OLGs would also cause an overall dysfunction in various signaling pathways. Because of a gain of tau functions, some signaling pathways would be abnormally depressed or activated; whereas because of a loss of tau function, selective signaling pathways would be negatively affected. The SH3-binding domain of tau has received attention. Such a domain binds to Fyn, phosphatidylinositol 3-kinase (PI3K), phospholipase C gamma 1 (PLC), and Grb2 [66,69]. These signaling pathways influence myelination, cell proliferation, and neuron-OLG interactions [76,77,78].

The consequences of tau on molecular chaperones is under extensive investigation. Molecular chaperones typically bind exposed hydrophobic residues of unfolded proteins by noncovalent interactions [79,80,81]. However, in CNS degenerative diseases, the proline-rich hydrophobic domains of tau are phosphorylated, and this phosphorylation interferes with the physiological processes that regulate protein levels [9,44].

Tau protein increases MT stability. In contrast, phosphotau, which is unable to bind the MTs, is detrimental to MT stability. Hyperphosphorylated non-MT-associated tau was found in CNS cells (neurons, OLGs, and astrocytes) of patients with primary progressive multiple sclerosis (MS) [82,83,84].

**Figure 2:**
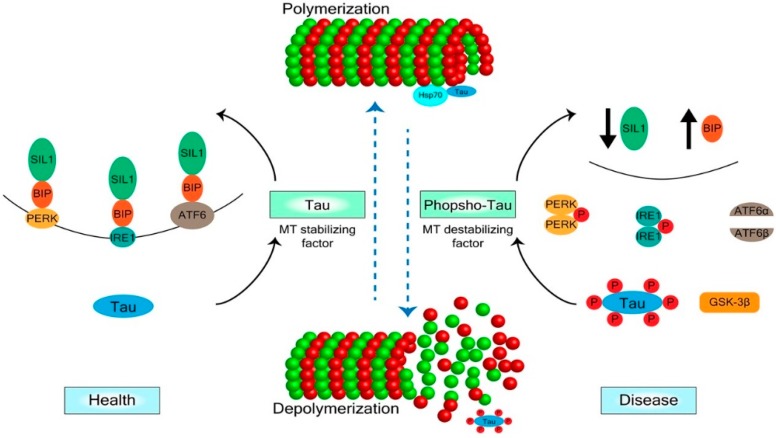
Mechanisms of Tau Regulation: Tau and the Endoplasmic Reticulum Stress Response during Progressive Degenerative Disorders of the CNS. Here we propose a model for tau-induced progressive degenerative disorders. Tau, microtubules (MTs), and the ER stress signaling are affected by each other. Tau stabilizes the MTs, whereas phosphorylated tau, MT instability, and the ER stress response progressively devastate CNS cells. The Bip chaperone frees sensor proteins (PERK, ATF, and IRE) that signal the inhibition of protein translation and promote apoptosis.

It is unknown how hyperphosphorylated tau implements progressive degenerative events. In neurons, non-MT-associated phosphotau and the endoplasmic reticulum (ER) chaperone proteins are known to reinforce each other during the emergence of progressive CNS diseases [10,80] (Figure 2). Both ER and cytosolic chaperones affect the regulation of phosphotau. Binding immunoglobulin protein (Bip), an ER chaperone, increases tau phosphorylation in neurons, ER-stress and phosphotau could potentiate each other in a vicious cycle [80], whereas SIL1, a co-chaperone that inhibits Bip, decreases tau hyperphosphorylation. Defective inhibition of Bip in the absence of SIL1 results in neurodegeneration [85]. In contrast, the cytosolic chaperones Hsp70 and Hsp90 bind to tau proteins [81,86], and elevated intracellular levels of these cytosolic chaperones promote tau binding to MTs and reduce tau phosphorylation. Decreased levels of these chaperones have the opposite effects on tau binding to MTs and tau phosphorylation (Figure 2).

When the ER stress response is not activated, Bip binds and inhibits protein kinase RNA-like ER kinase (PERK), inositol requiring enzyme 1a (IRE1a), and activating transcription factor 6 (ATF6) [87,88,89,90]. In contrast, when the ER stress response is activated, Bip releases PERK, IRE1a, and ATF6. In situations of prolonged unresolved stress, these signaling pathways suppress protein translation, promote neuronal apoptosis and demyelination [87,89,90,91] (Figure 2).

Bip deletion in OLGs decreases myelin, and the inactivation of Bip in OLGs results in OLG loss and severe myelin abnormalities [92,93]. One possibility is that a lack of Bip unleashes PERK, IRE1a, and ATF6, which results in protein synthesis suppression and cellular death.

Interestingly, activated PERK phosphorylates eIF-2α and causes a decrease in translation. Significantly elevated p-eIF-2α immunoreactivity has been observed in OLGs during experimental allergic encephalomyelitis, an animal model of MS [94], which further suggests an alteration of the ER stress pathway during this disease.

Phosphotau might carry out the demise of OLGs by deregulating protein levels. However, the precise deregulation of the chaperone machinery in the presence of phosphotau in OLGs is not completely understood. In this respect, much more work is needed. In summary, tau hyperphosphorylation has many deleterious consequences, and regulation of tau phosphorylation should be a preferred pharmacological target for neuroprotection.

## 3. Tau in Oligodendrocytes and CNS Degenerative Diseases

The group of CNS diseases with prominent tau pathology are named tauopathies [37,38,39,40,41,42,43]. Among this group, those with prominent tau pathology in OLGs include progressive supranuclear palsy, corticobasal degeneration, and frontotemporal dementia, which include Pick’s disease and some cases of Alzheimer’s disease [95,96,97,98,99]. These diseases have largely very poor therapeutic options; therefore, by understanding how a malfunction of tau in OLG starts CNS degeneration, rational approaches to treat these devastating diseases can be endorsed. Notably, some of these tauopathies reveal both myelin and gait anomalies, demonstrating the ability of myelin to influence axonal function. Gait abnormalities have been shown in corticobasal degeneration and in some clinical cases of frontotemporal lobar degeneration [96,98,99].

Despite the initial cause of the diseases, many CNS diseases result in widespread CNS degeneration. Of interest, relapsing-remitting MS ends with progressive CNS degeneration. Both mice with chronic experimental autoimmune encephalomyelitis, the animal model of MS, and tissues of patients with primary MS have hyperphosphorylated tau in CNS cells [15,82,83,84]. Progressive MS is characterized by gait abnormalities from the very onset of the disease [15,84]. Progressive MS is not considered a tauopathy for its pathogenesis; however, a dysfunction of tau in OLGs should be viewed as a driving force of progressive degenerative disorders of the CNS [15].

Overall, it is known that alterations of myelin affect neuronal functions [15] (Figure 1). In fact, tandem genomic duplication of the proteolipid protein 1 gene causes gait abnormalities and progressive myelin degeneration [100], and OLG phosphatase and tensin homolog are critical for both myelin and axonal integrity [101]. OLGs are believed to have a role in the pathogeneses of selective motor diseases [15]; recent work has indeed determined that OLG dysfunction has a role in the pathogenesis of amyotrophic lateral sclerosis, a neurodegenerative disease of motor neurons [102].

Several disorders that are characterized by movement anomalies are present in genetically proven, familial frontotemporal lobar degeneration. Notably, cases of familial frontotemporal lobar degeneration that are associated with granulin mutation have tau pathology in OLGs. Progranulin is a growth factor and progranulin reduction is associated with increased tau phosphorylation in P301L tau transgenic mice [103,104]. Staining for tau reveals neuronal pretangle forms and glial tau increase in both astrocytes and oligodendrocytes. Tau dysregulation in OLGs is believed to be partly responsible for these movement disorders. 

The proline-rich domain of OLG tau, which was previously shown to be critically important for in vivo myelination [15,19], is now a central focus of AD and other dementia. The Bridging INtegrator-1 (BIN1; also referred to as amphiphysin II and SH3P9) is a member of the Bin/Amphiphysin/Rvs (BAR) family of adaptor proteins that regulates membrane dynamics. Of great interest, BIN1’s SH3 domain interacts with tau’s proline-rich domain [105]. Predominant expression of AD-associated BIN1 occurs in mature oligodendrocytes and localizes to white matter tracts [105,106]. Consistent with the role of BIN1 in myelination, BIN1 expression increases at the onset of postnatal brain myelination and during differentiation of cultured oligodendrocytes [105,106]. In contrast, the loss of BIN1 significantly correlates with the extent of demyelination in MS lesions [105,106]. BIN1 undergoes complex alternative splicing to generate multiple isoforms that have diverse functions [105,106]. In this context, BIN-tau in OLGs may regulate myelin and neuronal health. 

In summary, myelin is believed to be the pillar on which healthy lifelong neuronal function can thrive [1,2,3,4,5,6]. Recent evidence has identified various myelin anomalies as an early sign for cognitive decline in dementia, Alzheimer’s disease, and other CNS degenerative diseases [107,108,109,110]. A key aspect of a healthy adult brain is the generation of new OLGs and internodes to improve the function of neurons [7,8]. Such lifelong myelination in the adult CNS requires a functional tau in OLGs in support of cellular extension, wrapping, and myelination. Further, it is currently unknown how neuron-specific regions differentially regulate OLG tau. Such differences must be understood in the context of how tau in OLGs promotes selective CNS degenerative diseases. The future challenge is to elucidate how tau in OLGs keeps the myelin capable of adapting to the ever-changing neuronal functions of the many regions of the CNS.
